# Colorimetric paper-based test strip for detection of methylparaben in nonconforming health care products

**DOI:** 10.1039/d6ra00484a

**Published:** 2026-05-11

**Authors:** Aya M. El-Hassanein, Sherin F. Hammad, Fotouh R. Mansour, Aya A. Abdella

**Affiliations:** a Department of Pharmaceutical Analytical Chemistry, Faculty of Pharmacy, Tanta University 31111 Egypt aya.atef.86@pharm.tanta.edu.eg; b Department of Medicinal Chemistry, Faculty of Pharmacy, King Salman International University (KSIU) Ras Sudr 46612 Egypt

## Abstract

Methylparaben (MPB) is a preservative used in pharmaceutical and food products to inhibit microbial growth and is also recognized as an endocrine disrupting compound. This work presents a simple, low cost, and reliable colorimetric paper based analytical device (PAD) for point of use detection of MPB in nonconforming health care products. The test strip was prepared by immobilizing Fe^3+^ on a chitosan modified paper substrate. Detection relies on the coordination interaction between MPB and the immobilized Fe^3+^, which produces a violet color on the paper surface. A smartphone was used to capture images of the test strip, and the images were analyzed using an RGB color detector mobile application. The intensity of the magenta component was extracted and used for quantitative analysis. The smartphone-based method showed strong correlation between magenta intensity and MPB concentration over the range of 10 to 50 mg mL^−1^ with a coefficient of determination (*R*^2^) of 0.989. The fabricated PAD demonstrated high accuracy, indicated by % recovery values ranging from 97.8 to 101.5, and precision with a relative standard deviation below 2 percent. The method was applied directly to determine MPB in health care products without any sample preparation. Statistical evaluation using *t*-test and *F*-test showed no significant difference between results obtained from the PAD and conventional colorimetric analysis. The developed approach enables rapid and practical on-site detection of nonconforming health care products.

## Abstract 2

Fe^3+^ immobilized on chitosan-modified cellulose paper was used as a test strip for rapid, cost-effective methylparaben quantification in pharmaceutical products *via* concentration-dependent color change and smartphone-based color analysis.

## Graphical abstract

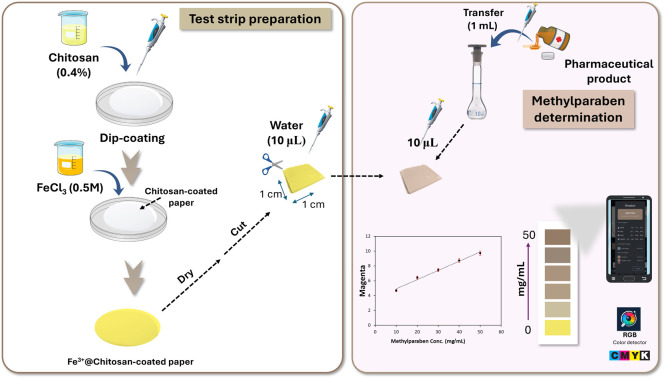

## 1 Introduction

Parabens, alkyl esters of *p*-hydroxybenzoic acid, have been used as preservatives in many foods, cosmetics, and pharmaceutical products due to their relatively low toxicity profile and long history of safe use.^1^ The antimicrobial activities of parabens seem to increase with increasing chain length. However, esters of longer alkyl chains are of limited application due to their lower solubility in water.^2^ Methylparaben (MPB), a methyl ester of *p*-hydroxybenzoic acid (IUPAC name: methyl 4-hydroxy benzoate), has been recognized among the most commonly used parabens.^3^ Owing to MPB potential endocrine-disrupting effects, the European Union legislation has set limits on MPB and its salts of up to 0.4% (w/w) in cosmetic and personal care products.^4^ Hence, the development of simple and reliable analytical methods to detect the presence of MPB beyond its allowed limits is crucial to ensure that products align with the regulation. Various methods and analytical techniques have been developed for determination of MPB, including high-performance liquid chromatography (HPLC), gas chromatography, electrophoresis, and spectrophotometry.^5^ Moreover, some electrochemical sensors have been developed for MPB determination utilizing an electrode modified with a cellulose nanocrystal–reduced graphene oxide nanocomposite^6^ or activated carbon/graphene oxide–gold nanoparticle nanocomposites,^7^ or a disposable screen-printed carbon-based electrode.^8^ These methods require sophisticated instruments, trained personnel, or special devices for readout, making them unsuitable for point-of-use testing (POUT).

POUT places analytical work at the site where samples originate or are being used. The rapidly appearing results of POUT support timely decisions in clinical care, environmental monitoring, and production control, reduce risks linked to storage and transport, and align testing speed with operational demands that depend on immediate evidence.^9^ POUT incorporates portable technologies and user-friendly interfaces, enabling non-specialist users to accurately conduct and interpret tests. POUT is often simple and inexpensive but sacrifices detection limit and operating range for sensitivity, specificity, or speed.^10–13^ The development of digital image colorimetry (DIC), which facilitated the use of peripheral technologies such as digital scanners, smartphone cameras, or other optical techniques, has helped in overcoming user variability while distinguishing color hue changes, improving accuracy of quantitation, and maintaining the desired reproducibility.^14–16^

The emergence of DIC has revolutionized the development of colorimetric methods. Regarding MPB, Ko *et al.*^17^ has fabricated a finger pump microfluidic system for detection of MPB in food samples that relied on a modified Fenton reaction at 40 °C to form a green complex. The sum of Red (R) + Green (G) value of the RGB model, detected using a smartphone, was linearly correlated to MPB concentration. This system, despite being portable and easy to use, it suffers from numerous limitations, including the potential toxic effects and environmental hazards of the reactive oxygen species generated from the Fenton reaction and unsustainability.^17^ The linearity range of the reported smartphone-based method is 0.1–3.0 mg mL^−1^, which is below the permissible MPB limits. As a result, it is not functional for POUT to identify nonconforming products.^17^ Thus, a simple affordable and non-toxic approach that can discriminate between the conforming and nonconforming products, based on the MPB content is required.^18^

Paper-based analytical devices (PADs) represent a growing class of chemical sensor technologies designed for POUT applications. PADs fabrication entails the deposition or immobilization of specific reagents on paper or surface modified paper substrate, respectively. Simple reagent deposition is restricted to hydrophobic reagents and sensor elements.^19^ On the other hand, reagent immobilization is preferred for the hydrophilic reagents, where colorimetric indicator is fixed on a hydrophobic matrix of coated paper surface.^20^ PADs, when coupled with smartphone detection, are portable and less resource intensive compared to classical approaches that rely on the use of peripheral equipment for quantitative measurement.^21^ Moreover, PADs, which have been fabricated and used so far, are single-use or disposable, eliminating the memory effect and reducing the risk of cross-contamination.^22^

Chitosan (CS), derived from chitin, is a biocompatible, bioactive, and biodegradable biopolymer that has been widely applied in the field of analysis and aligns with the greenness and sustainability aspects.^23^ CS received a lot of attention in several areas, such as carbonized polymer dots,^24^ chiral separation,^25,26^ solid phase extraction,^27–29^ and PADs,^30,31^ due to its abundant chemical functionalities, good film-forming capabilities, and easy crosslinking. CS is a viable option for paper surface modification in PADs because of its ecological sustainability and active functional hydroxyl and amino group, and metal complexing ability.^32^

Color analysis can be conducted using various software applications installed on the smartphone, allowing for the extraction of a suitable color component response that is dependent on analyte concentration. Various color models are discussed in the literature, such as RGB, CMYK, and HSL.^31^ The selection of an appropriate color model and component largely depends on the specific shade developed.^33,34^ Color intensities derived from the coordinates of various color spaces are utilized to determine analyte concentration through the capture of diffuse reflectance images.^35^ An image of the sample can be correlated with the complementary color resulting from the chemical reaction. This image can be captured using a smartphone, camera, webcam, or tablet.^36^ Chemical analysis using a smartphone is particularly advantageous due to its portability,^37^ cost-effectiveness,^38^ and user-friendliness.^39^

In this work, a simple, sustainable, ecofriendly, and cost-effective colorimetric paper-based test strip was fabricated, validated, and successfully applied for POUT of MPB in health care products. The developed PAD is based on the coordination interaction between MPB and Fe^3+^ ions immobilized on a paper substrate *via* CS surface modification. The underlying chemical reaction was studied and statistically compared to the PAD/smartphone method using both *t*-test and *F*-test, revealing no significant differences. Unlike the reported analytical methods, the developed test strip is considered simple, affordable, rapid, and does not require either tedious procedure or sophisticated and expensive instruments. To the best of our knowledge, this is the first report of a PAD for POUT of MPB.

## 2 Materials and methodology

### 2.1 Chemicals and materials

MPB was supplied by Pharmalog (China). Absolute ethanol (HPLC) (99.8%), methanol (HPLC), acetone (HPLC), CS (medium MW, deacetylation degree 75–85%), and acetic acid (99.9%) were purchased from Sigma-Aldrich (Missouri, USA). Ethyl ether Anhydrous (HPLC) was purchased from TEDIA COMPANY (USA). Anhydrous ferric chloride was supplied by SRL Chemicals (Mumbai, India). Double-ring filter paper 102 (9.0 cm diameter) was used for the test strip preparation (China). Sample products, Zyrtec^®^ drops manufactured by GlaxoSmithKline (Egypt) and Injectmol^®^ vial produced by PHARCO B International (Egypt), were purchased from a community pharmacy. All the chemical reagents were used without further purification.

### 2.2 Instruments

UV/Vis spectra were recorded in the range of 400–800 nm using a Jasco V-530 UV/Vis double beam spectrophotometer (Tokyo, Japan). A DAIHAN hotplate magnetic stirrer (Batam, Indonesia) was used. All the materials were weighed using a Sartorius BP221S 4-digit analytical balance (Göttingen, Germany). BRANSONIC (3510) ultrasonic cleaner (Danbury, USA). Hettich centrifuge (Tuttlingen, Germany). Scanning electron microscopy (SEM) images and energy-dispersive X-ray (EDX) analysis were acquired using JOEL JSM IT-00 scanning electron microscope (Tokyo, Japan). A smartphone equipped with a 12-megapixel camera and an Ios 17.6.1 operating system (iPhone 11, Vietnam). Color analysis was performed using the freely available application of RGB color detector (version 11.2) from the app Store. For static water contact angle (WCA) measurements, a smartphone setup was utilized for photo acquisition.^40^ Photos of a 100 µL water drop were acquired using single take mode (15 s), transferred to a PC and processed using Image J 1.54g (version 1.8.0_345).^29^ WCA measurement was conducted using Drop_ analysis Plug-In ref. 41, as follows: Run ImageJ, open an image, image → type → 32-bit, plug-in → drop_ and analysis → drop analysis-LB-ADSA.

### 2.3 Test strip preparation

**Scheme 1:**
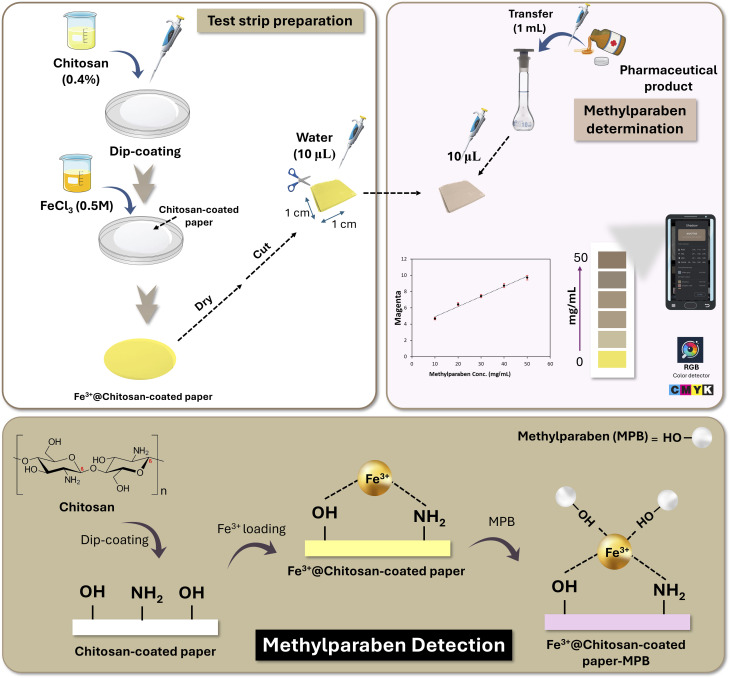
Diagrammatic representation of test strip preparation and smartphone-based determination of methylparaben (MPB), showing the proposed recognition and detection events.

For the preparation of PAD, the filter paper was soaked in 5 mL of 0.4% CS and left to dry completely at room temperature.^29,30^ Then, 2 mL of 0.1 M FeCl_3_ was placed on the CS-coated paper and allowed to dry at room temperature. Finally, the prepared paper was then cut into small square pieces (1 × 1 cm) and stored in a dry clean container until further use (Scheme 1).

### 2.4 General procedure for MPB determination

A stock standard solution of 100 mg mL^−1^ MPB was prepared in ethanol and diluted using the same solvent to obtain working standard solutions ranging between 3 and 50 mg mL^−1^. For PAD/smartphone measurements, the test strip was wet with 10 µL water. Then, a volume of 10 µL of working standard MPB solutions (10–50 mg mL^−1^) was added. Photographs were acquired with a smartphone in a white walled imaging box analyzed using RGB color detector application to extract color components. The intensity of the magenta color (*M*) was plotted against the corresponding MPB concentration (mg mL^−1^).

For colorimetric measurements, in a 5 mL volumetric flask, 0.5 mL of freshly prepared 0.5 M aqueous FeCl_3_ was mixed with 0.5 mL of working MPB standards (3–50 mg mL^−1^). The solution was made up to the total volume using distilled water. The absorbance was recorded at 528 nm and plotted against the corresponding MPB concentration (mg mL^−1^) to construct the calibration curve.

### 2.5 Sample application

The developed PAD was applied for MPB detection in different pediatric pharmaceutical products, Zyrtec® drops and Injectamol® vial. In a series of 10 mL volumetric flasks, 1 mL of each sample was mixed with 1 mL of standard MPB (0–40 mg mL^−1^). The solution was made up to final volume with distilled water. An aliquot of 10 µL of this mixture was added to the test strip, previously wet with 10 µL water. Photos were acquired and analyzed as aforementioned in Section 2.4. MPB concentration was calculated from the constructed calibration curve (mg mL^−1^) for both PAD/smartphone and colorimetric methods.

## 3 Results and discussion

**Fig. 1:**
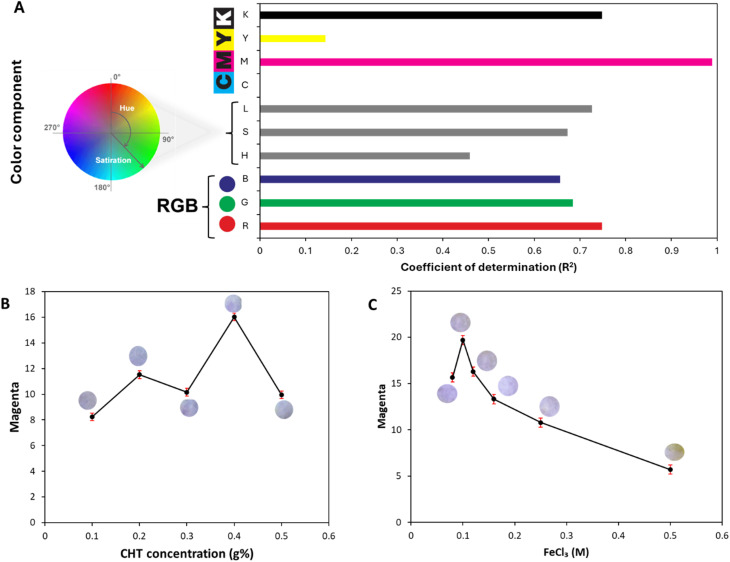
Optimization of paper-based test strip preparation conditions: (A) color model selection, (B) effect of chitosan concentrations (*g*%), (C) effect of Fe^3+^ concentrations (*M*) (error bars represent ±SD of three replicate determinations).

It is anticipated that MPB, being a phenolic molecule, will combine with Fe^3+^, in presence of water to form a violet-colored complex.^42^ In this study, a paper-based test strip was designed and fabricated for MPB determination relying on its ability to complex immobilized Fe^3+^ on amine-functionalized CS-modified paper surface, changing the paper surface from light yellow into violet (Scheme 1). The developed color on test strip was detected using a smartphone and different color components were extracted. The different color components of the RGB, HSL, and CMYK models were extracted. The color component was selected based on the coefficient of determination (*R*^2^), where the magenta (*M*) component of the CMYK model showed the largest *R*^2^, indicating the best correlation to MPB concentration (Fig. 1A). Moreover, the conditions for the colorimetric determination of MPB were also established and used as a control for testing the reliability of the smartphone detection using the developed test strip.

### 3.1 Paper-based test strip for MPB determination

#### 3.1.1 Test strip preparation and characterization

Paper sheets were coated with CS so that Fe^3+^ could be chemically adsorbed on its surface instead of physical entrapment on uncoated paper to afford PAD stability. To confirm the importance of CS in the developed test strip, Fe^3+^ was applied to the paper surface without any CS coating. In the absence of CS, Fe^3+^ was rapidly lost upon wetting, resulting in damage to the test strip and a failure to develop the desired color upon the addition of MPB. This observation underscored the critical role of CS in the effectiveness of the developed test strip.

The effect of concentration of CS coating solution and Fe^3+^ solution were studied to find the optimum conditions that would achieve the highest M intensity. CS coating solutions of concentrations ranging between 0.1 and 0.5% were tried (Fig. 1B). It could be noticed that the intensity of the developed color was directly proportional to CS concentration reaching its max at 0.4%. Using 0.5% CS, the color intensity was markedly diminished, which can be ascribed to the reduced wettability reported with high CS concentrations.^29^ Also, different Fe^3+^ concentrations (0.08–0.5 M) were studied. As shown in Fig. 1C, 0.1 M Fe^3+^ was sufficient to produce clear violet color with minimum yellow color in the background. Apparently, the strong yellow color observed at higher Fe^3+^ concentrations obscured the appearance of a clear violet tinge. Accordingly, the test strip was prepared using 0.4% CS and 0.1 M Fe^3+^ solutions.

**Fig. 2:**
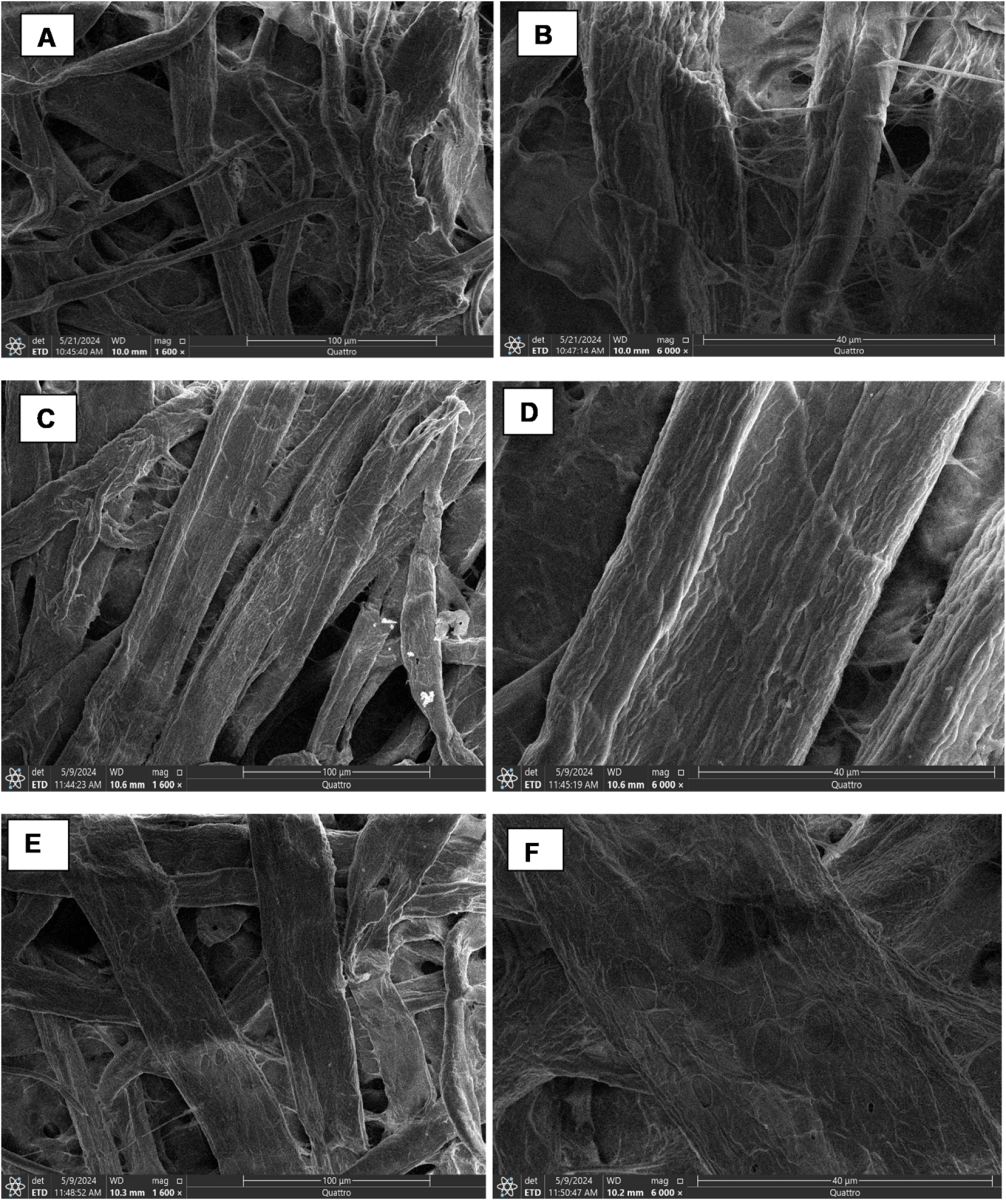
Scanning electron micrographs of uncoated paper (A and B) and CS-coated paper (C and D) Fe^3+^@CS-coated paper (E and F) at 100 µm and 40 µm.

The prepared test strip was characterized using SEM, EDX-SEM, and WCA measurements. SEM and SEM-EDX were applied to inspect PAD surface morphology and elemental composition, respectively. The WCA is an approach to evaluate surface wettability and hydrophobicity, where larger WCA values indicate more hydrophobicity.^31^ The WCA of the CS-coated and Fe^3+^@CS-coated paper were found to be 76.68° ± 1.2 and 43.43° ± 0.9, respectively. SEM micrographs offer important details on the morphological alterations brought on by surface modifications. The uncoated paper showed irregular fibrous surface with numerous irregular voids, a characteristic morphology of the cellulose-based substrates, as depicted in Fig. 2A and B. Conversely, the CS-coated paper (Fig. 2C and D) showed less voids, indicating CS deposition on paper's surface,^31^ which was augmented by a marked decrease in paper's wettability (WCA = 76.68° ± 1.2) (Fig. S1). Further modification with Fe^3+^, shown in Fig. 2E and F, did not show an obvious difference from the CS-coated paper. However, the effectiveness of Fe^3+^ immobilization was confirmed by the significant decline in the WCA from 76.68° ± 1.2, for CS-coated to 43.43° ± 0.9 after Fe^3+^ fixation (Fig. S1). The Fe^3+^@CS-coated paper showed an observed increase in surface hydrophilicity that could be ascribed to the ionic character of Fe^3+^, which can enhance water affinity at the surface interface (Fig. S1).^43^ This indicated that the incorporation of Fe^3+^ into the CS matrix not only modifies the chemical composition but also alters the surface energy in a manner that enhances wettability. The elemental composition was further determined using EDX-SEM. The uncoated paper surface showed an elemental composition of C (43.89%) and O (56.11%) (Fig. S2A), which was changed to C (51.58%), O (39.39%), and N (9.03%) after coating with CS (Fig. S2B). This could be ascribed to presence of nitrogen-rich amino groups of CS. Moreover, the Fe^3+^@CS-coated paper showed C (38.49%), O (54.92%), N (5.78%), Fe (0.27%), and Cl (0.54%) (Fig. S2C). The EDX-SEM analyses collectively confirm successful surface modification and chemical incorporation of CS and its Fe^3+^-treated variant. Overall, the progressive transition from uncoated to Fe^3+^@CS-coated paper confirm effective surface modification, which is likely to influence the paper's physical, chemical, and functional properties in downstream analytical applications.

#### 3.1.2 Determination of analytical performances of the test strip

**Fig. 3:**
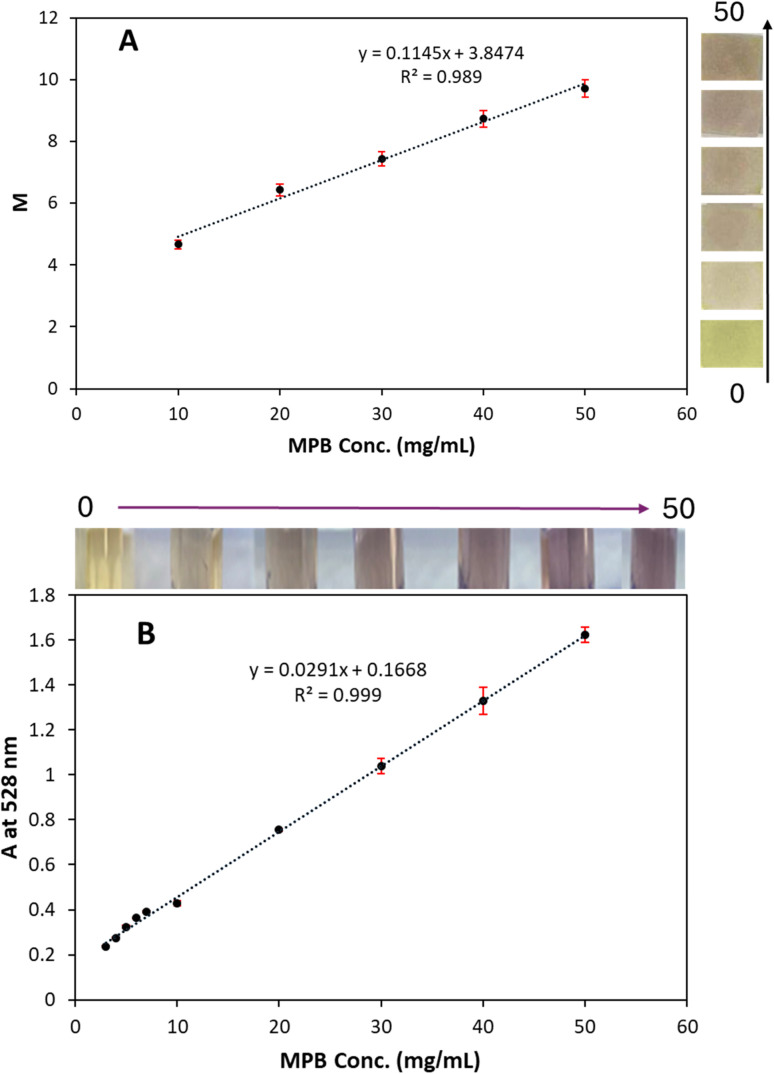
Linearity results for MPB determination using the developed Fe^3+^@CS test strip (10–50 mg mL^−1^) (A) and colorimetric method (3–50 mg mL^−1^) (B) (inset photos: test strip and colored solutions with MPB).

The developed test strip was applied for MPB detection in standard solution. The test strip could develop the violet color as a response to the presence MPB only when prewet with water. This could be ascribed to the contribution of water molecules in the formed complex.^30^ The magenta (*M*) component of the CMYK color model showed an observed dependence on MPB concentration (*R*^2^ = 0.989), indicating good linearity (Fig. 3A). The developed strategy was validated according to the ICH-Q2 guidelines. In terms of linearity, the response was found to be linear between 10–50 mg mL^−1^. Regression parameters were calculated and are presented in Table 1. The limit of detection (LOD) was found to be 1.1 mg mL^−1^, indicating the ability of the method to detect MPB beyond the acceptable limits (4.0 mg mL^−1^). The accuracy of the proposed strip was evaluated using the recovery results at three different concentration levels, 20, 30, and 40 mg mL^−1^. As presented in Table S1, accuracy was indicated by the mean percentage recovery within 100.00% ± 2% at all concentration levels. Moreover, both intraday and inter-day precision were indicated by a %RSD of less than 2% for all concentrations, as presented in Table S2.

**Table 1:** Regression parameters for the determination of MPB using the proposed test strip/smartphone and colorimetric methods

Parameters	Test strip/smartphone	Colorimetry
Linearity range (mg mL^−1^)	10–50 mg mL^−1^	3–50 mg mL^−1^
Coefficient of determination (*R*^2^)	0.989	0.999
Slope ± SD^a^	0.11 ± 0.009	2.9 ± 0.03 × 10^−2^
Intercept ± SD^a^	3.85 ± 0.32	1.6 ± 0.07 × 10^−1^
Residual SD^a^	0.31	0.016
LOD^b^ (mg mL^−1^)	1.10	0.88

a Standard deviation.

b Limit of detection.

### 3.2 Colorimetric determination of the MPB–Fe^3+^ complex

**Fig. 4:**
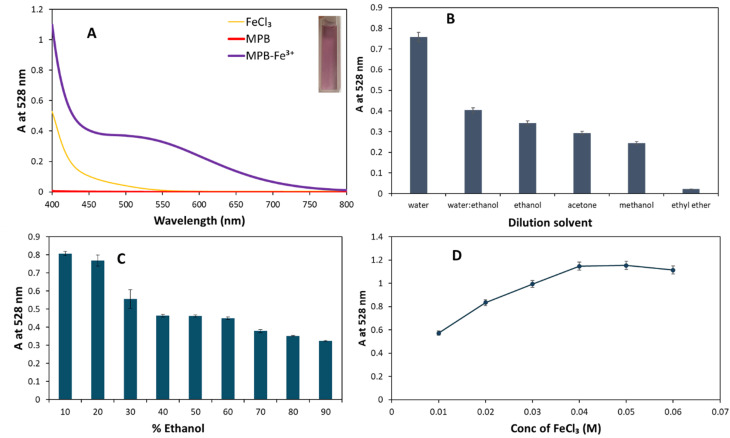
Optimization of MPB–FeCl_3_ reaction conditions (A) absorption spectra of MPB, FeCl_3_, and MPB–Fe^3+^ complex, (B) effect of dilution solvent, (C) effect of ethanol percentage in the reaction media, and (D) effect of FeCl_3_ concentration (error bars represent ±SD of three replicate determinations).

Upon the addition of MPB to FeCl_3_, a violet-colored solution was produced due to the formation of MPB–Fe^3+^ complex (Fig. 4A: inset photo). The UV-Vis spectrum of the formed complex exhibited a distinct absorbance peak at 528 nm (Fig. 4A). The optimum conditions for the formation of MPB–Fe^3+^ complex were determined. The studied factors included dilution solvent and FeCl_3_ concentration. Different dilution solvents were tried to find out the best solvent system for the formation of a stable MPB–Fe^3+^ complex. Water produced the highest absorbance, followed by water–ethanol mixtures and pure ethanol (Fig. 4B). The presence of water was found essential for the complex formation that was ascribed to the participation of two water molecules in the coordination complex.^30^ The decrease in absorbance in organic solvents such as acetone, methanol, and ethyl ether suggest a reduced solubility or interaction efficiency of the chromogenic complex in less polar environments. This highlights the critical role of solvent polarity in assay sensitivity as shown in (Fig. 4C). Increasing ethanol concentration leads to a gradual decline in absorbance, suggesting that high ethanol content disrupts the chromogenic reaction, possibly due to altered solvent polarity or reduced aqueous compatibility. This supports the preference for aqueous or mixed aqueous media. Accordingly, water is necessary for the complex formation reaction which accounts for the necessary wetting of test strip.

Furthermore, the effect of FeCl_3_ concentration was investigated (0.01 to 0.0625 M) (Fig. 4D). A saturation trend was observed with increasing FeCl_3_ concentration, where absorbance increases up to 0.04 M and then plateaus, indicating the formation of a stable complex. The concentration of FeCl_3_ was maintained at 0.05 M to ensure good reproducibility and robustness.

The colorimetric strategy showed very good linearity (*r* = 0.999) over a concentration range from 3–50 mg mL^−1^. A good correlation was indicated by a coefficient of determination (*R*^2^) of 0.999, as shown in Fig. 3B. Regression parameters were calculated and are presented in Table 1. The proposed strategy showed excellent accuracy and precision, as presented in Tables S1 and S2, respectively.

### 3.3 Sample application

The developed test strip was applied for determination of MPB in health care products. Since the concentration of MPB in the tested products was lower than method's LOD and because of the diverse nature of sample matrices that were difficult to regenerate for anti-interference testing, spiked samples were analyzed to test the selectivity and detect any potential interferences form sample matrix (Table 2). The obtained percentage recovery values demonstrated the selectivity of the method (Table 2). Also, the samples were analyzed colorimetrically at 528 nm. Both *t*-test and *F*-test were performed to statistically compare test-strip/smartphone and colorimetric methods' results. No statistically significant differences were observed between the results obtained by both methods (Table 2). This indicated that the developed test strip could successfully be used for the determination of MPB content in different products with good accuracy and precision using simple procedures, a small number of reagents, and short preparation and reaction times. Additionally, the developed PAD required neither sophisticated nor expensive instruments.

**Table 2:** Assay results for determination of drug samples using the devised paper-based test strip/smartphone and colorimetric methods

Sample	Method								*F*-Test^a^	*t*-Test^a^
	Test strip/smartphone				Colorimetry					
	Conc. taken (mg mL^−1^)	Mean found conc. (mg mL^−1^) ± SD (*n* = 3)	Mean % recovery ± SD	%RSD	Conc. taken (mg mL^−1^)	Mean found conc. (mg mL^−1^) ± SD (*n* = 3)	Mean % recovery ± SD	%RSD		
1	20	21.13 ± 1.0	105.50 ± 5.02	4.75	20	21.10 ± 0.10	105.20 ± 0.52	0.47	0.30	0.52
	30	28.40 ± 0.51	95.30 ± 1.60	1.68	30	32.21 ± 0.18	107.30 ± 0.64	0.60		
	40	41.70 ± 2.02	104.30 ± 5.07	4.85	40	41.61 ± 0.51	102.75 ± 1.25	1.22		
2	20	18.80 ± 0.43	91.50 ± 2.17	2.37	20	20.17 ± 0.25	100.8 ± 1.25	1.16	0.09	0.38
	30	29.70 ± 1.36	99.20 ± 4.50	4.64	30	29.52 ± 0.95	98.33 ± 3.21	3.27		
	40	38.7 ± 0.47	96.8 ± 1.2	1.24	40	39.47 ± 0.21	98.67 ± 0.52	0.53		

a *p*-Value.

Moreover, the developed test strip exhibited a linearity range that matches MPB concentrations in nonconforming products. Thus, it can be easily used by end users for the POUT. However, yellow-colored solutions (dosage forms containing dyes) were found to obscure the formed violet color, which requires the sample clean up *via* ion exchange solid phase extraction. In addition, the test strip cannot be applied to salicylate containing drug products.

## 4 Conclusion

A simple and effective paper based analytical device (PAD) was developed for the detection of methylparaben in health care products. The device is based on Fe^3+^ immobilization on a chitosan modified paper substrate. The interaction between methylparaben and Fe^3+^ produces a distinct violet color that enables visual and quantitative analysis. Smartphone based color analysis provided reliable quantification. The magenta color component of the CMYK color model showed a strong linear correlation with methylparaben concentration in the range of 10 to 50 mg mL^−1^. Good analytical performance was achieved with accuracy between 97.8 and 101.5 percent and precision (RSD < 2%). Application to real health care samples confirmed method reliability in comparison to the photometric measurements. Statistical comparison using both *F*-test and *t*-test shows no significant difference from conventional colorimetric method. The proposed PAD offers a low cost and portable alternative for routine methylparaben monitoring in nonconforming products. The method supports rapid screening and on-site analysis without the need for specialized instrumentation.

## Author contributions

Aya M. El-Hassanein: methodology, investigation, validation, writing original draft, Sherin F. Hammad: supervision, review and editing, Fotouh R. Mansour: supervision, review and editing, Aya A. Abdella: conceptualization, formal analysis, methodology, investigation, validation, writing – review and editing.

## Conflicts of interest

The authors declare no competing interests.

## Supplementary Material

RA-016-D6RA00484A-s001

## Data Availability

The data supporting this article have been included as part of the supplementary information (SI). Supplementary information is available. See DOI: https://doi.org/10.1039/d6ra00484a.

## References

[cit1] Tavares R. S., Martins F. C., Oliveira P. J., Ramalho-Santos J., Peixoto F. P. (2009). Reprod. Toxicol..

[cit2] Xie Y.-T., Chen P., Wei W.-Z. (1999). Microchem. J..

[cit3] Maeda Y., Yamamoto M., Owada K., Sato S., Masui T., Nakazawa H. (1989). J. Assoc. Off. Anal. Chem..

[cit4] Byford J. R., Shaw L. E., Drew M. G. B., Pope G. S., Sauer M. J., Darbre P. D. (2002). J. Steroid Biochem. Mol. Biol..

[cit5] Mohd Rudi M. F. (2020). Def. S T Tech. Bull..

[cit6] Wan Khalid W. E. F., Mat Arip M. N., Jasmani L., Lee Y. H. (2019). Sensors.

[cit7] Malarat N., Oin W., Kanjana K., Makkliang F., Siaj M., Poorahong S. (2023). Microchem. J..

[cit8] Lucas-Sánchez S., Abad-Gil L., Isabel-Cabrera C., Gismera M. J., Sevilla M. T., Procopio J. R. (2022). Talanta.

[cit9] Schubert A., Harrison J., Kent-Buchanan L., Bonds V., McElmurry S. P., Love N. G. (2024). Sci. Data.

[cit10] Barnes S., Scornavacca E. (2004). Int. J. Mobile Commun..

[cit11] Clarke S. F., Foster J. R. (2012). Br. J. Biomed. Sci..

[cit12] Korhonen I., Pärkkä J., van Gils M. (2003). IEEE Eng. Med. Biol. Mag..

[cit13] Martinez A. W., Phillips S. T., Carrilho E., Thomas 3rd S. W., Sindi H., Whitesides G. M. (2008). Anal. Chem..

[cit14] Songjaroen T., Dungchai W., Chailapakul O., Henry C. S., Laiwattanapaisal W. (2012). Lab Chip.

[cit15] Ellerbee A. K., Phillips S. T., Siegel A. C., Mirica K. A., Martinez A. W., Striehl P., Jain N., Prentiss M., Whitesides G. M. (2009). Anal. Chem..

[cit16] Jokerst J. C., Adkins J. A., Bisha B., Mentele M. M., Goodridge L. D., Henry C. S. (2012). Anal. Chem..

[cit17] Ko C.-H., Liu C.-C., Huang K.-H., Fu L.-M. (2023). Food Chem..

[cit18] Rink S., Baeumner A. J. (2023). Anal. Chem..

[cit19] Consonni V., Baccolo G., Gosetti F., Todeschini R., Ballabio D. (2021). Chemom. Intell. Lab. Syst..

[cit20] Płotka-Wasylka J., de la Guardia M., Andruch V., Vilková M. (2020). Microchem. J..

[cit21] Cate D. M., Dungchai W., Cunningham J. C., Volckens J., Henry C. S. (2013). Lab Chip.

[cit22] Shojaeifard Z., Hemmateenejad B. (2022). Sens.
Actuators, B.

[cit23] Mabrouk M., Hammad S. F., Mansour F. R., Abdella A. A. (2024). Crit. Rev. Anal. Chem..

[cit24] Abdella A. A., Hammad S. F. (2024). Microchem. J..

[cit25] Mabrouk M., Hammad S. F., Abdella A. A., Mansour F. R. (2022). Int. J. Biol. Macromol..

[cit26] Mabrouk M., Hammad S. F., Abdella A. A., Mansour F. R. (2024). Chromatographia.

[cit27] Abdella A. A., Ulber R., Zayed A. (2019). Carbohydr. Polym..

[cit28] Shehabeldeen M. T., Mansour F. R., El-Malla S. F., Abdella A. A. (2025). Talanta Open.

[cit29] El-Zayat E., Hammad S. F., El-Malla S. F., Abdella A. A. (2025). J. Chromatogr. A.

[cit30] El-Hassanein A. M., Mansour F. R., Hammad S. F., Abdella A. A. (2024). RSC Adv..

[cit31] Abdella A. A., Elshenawy E. A. (2025). Talanta.

[cit32] Mabrouk M., Hammad S. F., Abdella A. A., Mansour F. R. (2021). Colloids Surf., A.

[cit33] Abdella A. A., Ulber R. (2025). Microchem. J..

[cit34] Ansari N., Lodha A., Pandya A., Menon S. K. (2017). Anal. Methods.

[cit35] Pessoa K. D., Suarez W. T., Dos Reis M. F., de Oliveira Krambeck Franco M., Moreira R. P. L., Dos Santos V. B. (2017). Spectrochim. Acta, Part A.

[cit36] Benedetti L. P. d. S., dos Santos V. B., Silva T. A., Filho E. B., Martins V. L., Fatibello-Filho O. (2015). Anal. Methods.

[cit37] Gaballah A. Y., Hammad S. F., Mansour F. R., Abdella A. A. (2025). Microchem. J..

[cit38] Minh-Huy D., Anh-Dao L.-T., Thanh-Nho N., Nhon-Duc L., Cong-Hau N. (2023). Food Chem..

[cit39] Martins L., De Souza Silva A., Moraes L., Godoy B., Gonçalves I., Rocha F. (2020). Proc. – U. S. Nav. Inst..

[cit40] Chen H., Muros-Cobos J. L., Amirfazli A. (2018). Rev. Sci. Instrum..

[cit41] Stalder A. F., Melchior T., Müller M., Sage D., Blu T., Unser M. (2010). Colloids Surf., A.

[cit42] Vucane S., Cinkmanis I., Juhnevica-Radenkova K., Sabovics M. (2024). Foods.

[cit43] Hebbar R. S. , Isloor A. M. and Ismail A. F., in Membrane Characterization, Elsevier, 2017, pp. 219–255

