# Supplementary material for: Colorimetric paper-based test strip for detection of methylparaben in nonconforming health care products
Source: RSC Adv. 2026 May 11;16(27):24752–60. doi: 10.1039/d6ra00484a (PMC13159625; doi:10.1039/d6ra00484a)
Supplement: RA-016-D6RA00484A-s001 [file RA-016-D6RA00484A-s001.pdf]

Supplementary Information (SI) for RSC Advances.
This journal is © The Royal Society of Chemistry 2026

Supplementary material

Colorimetric Paper-Based Test Strip for Detection of Methylparaben in Nonconforming Health Care Products

Aya M. El-Hassanein[1], Sherin F. Hammad[1], Fotouh R. Mansour[1,2], and Aya A. Abdella[1]*

[1]Department of Pharmaceutical Analytical Chemistry, Faculty of Pharmacy, Tanta University, 31111, Egypt.
[2] Department of Medicinal Chemistry, Faculty of Pharmacy, King Salman International University (KSIU), Ras Sudr 46612, Egypt

* Correspondence to Aya A. Abdella

aya.atef.86@pharm.tanta.edu.eg, Aya.atef.86@gmail.com

	A	B
**Drop shape**	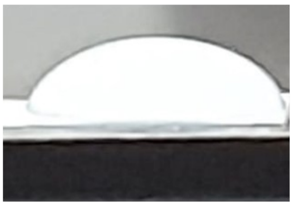	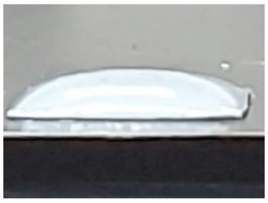
**WCA**	**76.68°**	**43.43°**

Fig. S1. Photos and water contact angle (WCA) of CS-coated paper (A) and Fe^{3+}@CS-coated paper (B) using 100 μL distilled water.

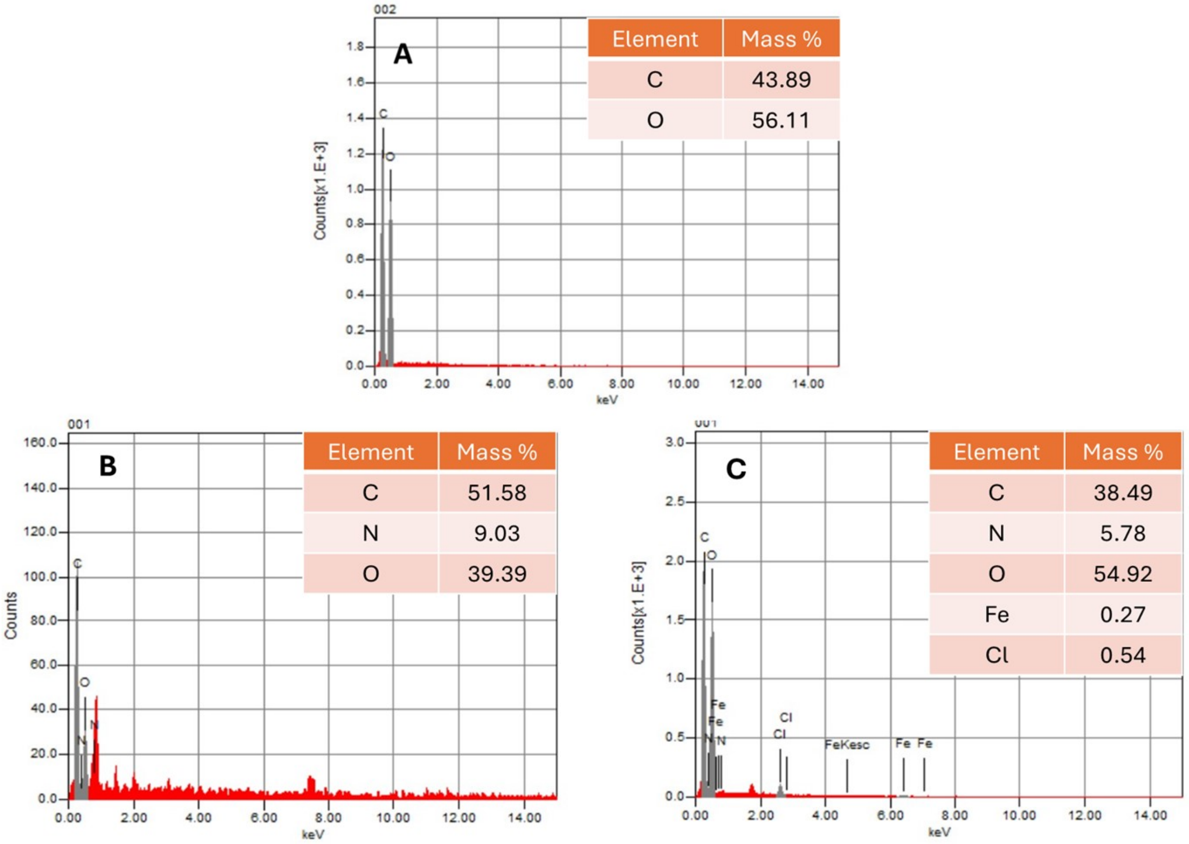

Fig. S2. EDX-SEM spectra of (A) uncoated paper and (B) CS-coated paper (C) Fe^{3+}@CS-coated paper.

Table S1. Accuracy of the proposed test strip/ smartphone and colorimetric method for determination of the MPB

Method	Conc. added (mg/mL)	Conc. found (mg/mL) (n=3)	% recovery	Mean % recovery ± SD
**Test strip/ Smartphone**	30	32.01	106.7	101.44 ±4.56
		29.66	98.86	
		29.63	98.76	
	40	39.44	98.6	97.80 ± 0.69
		38.96	97.4	
		38.96	97.4	
	50	48.98	97.96	100.86 ±2.50
		50.97	101.97	
		51.33	102.66	
**Colorimetry**	25	25.04	100.16	100.45 ±0.79
		24.96	99.84	
		25.34	101.36	
	35	35.29	100.83	99.66 ± 1.15
		34.49	98.54	
		34.87	99.63	
	45	45.11	100.24	100.31 ±0.64
		45.44	100.98	
		44.87	99.71	

Table S2. Intra- and inter-day precision results for determination of MPB using the proposed test strip/ smartphone and colorimetric methods

**Method**	**Conc. added (mg/mL)**	**Intraday precision**			**Inter-day precision**		
		Conc. found (mg/mL) ±SD (n=9)	(mg/mL) mean recovery ± SD	%RSD	Conc. found ±SD (n=9)	% Mean recovery ± SD	%RSD
**Test strip/ smartphone**	30	30.43±1.36	101.44±4.55	4.55	29.97±1.49	99.91±1.35	1.36
	40	39.12±0.27	97.8 ± 0.69	0.69	40.04±1.96	100.10±2.05	2.05
	50	50.42±1.26	100.86±2.53	2.53	49.97±1.40	99.95±0.81	0.81
**Colorimetry**	25	25.11±1.03	100.45±0.79	0.79	25.04±0.24	100.16±0.24	0.25
	35	34.88±0.94	99.66±1.15	1.15	34.88±0.31	99.68 ± 0.29	0.29
	45	45.14±1.22	100.31±0.64	0.64	45.04±0.52	100.10±0.17	0.18
